# A Hispanic Female Presenting With the Cultural Syndrome “El Salto” as a Psychosomatic Symptom of Anxiety

**DOI:** 10.7759/cureus.92861

**Published:** 2025-09-21

**Authors:** Jessica Barcelo, Emilio Blair, Raphael Crespo, Sakhi Shah, Patricia Junquera, Ashley Matejka

**Affiliations:** 1 Psychiatry and Behavioral Sciences, Florida International University, Herbert Wertheim College of Medicine, Miami, USA; 2 Research and Development, QHSLab, West Palm Beach, USA

**Keywords:** anxiety, cultural competency, cultural syndrome, geriatric psychiatry, hispanic or latino, salto

## Abstract

“El salto” is a culturally embedded somatic expression of distress generally experienced as a sudden “jump” or churning in the upper abdomen. This phenomenon is common in Spanish-speaking Caribbean communities but is not formally recognized in the Diagnostic and Statistical Manual of Mental Disorders, Fifth Edition (DSM-5); however, it parallels Hispanic cultural syndromes such as *ataque de nervios*, *nervios*, and *susto*. The present case describes an 87‑year‑old Cuban‑American woman with newly diagnosed Parkinson's disease who presented with moderate depression and anxiety assessed via Patient Health Questionnaire‑9 (score of 12) and General Anxiety Disorder‑7 (score of 11) and lifelong episodes of “el salto” during stress, which she and her community viewed as a normative cultural experience rather than a psychiatric symptom. Only through detailed questioning and the use of the Cultural Formulation Interview (a structured cultural psychiatric interview tool provided in the DSM-5) did she reveal that “el salto” had framed her understanding of distress since adolescence. Integration of her explanatory model with biopsychosocial insights led to a diagnosis of generalized anxiety disorder and major depressive disorder, and a treatment plan of escitalopram and bupropion was initiated. This case underscores how “el salto,” shaped by linguistic relativity and the influence of cultural norms on somatic expression, can delay recognition of treatable mood and anxiety disorders. Clinicians should routinely elicit culturally specific idioms of distress to avoid misdiagnosis, improve rapport, and deliver culturally responsive care. Future research should validate “el salto” across broader Hispanic cohorts and develop tailored screening tools.

## Introduction

The Hispanic population includes individuals who identify with Hispanic heritage, whether through cultural or ethnic affiliation, ancestry from Latin America or the Caribbean, or origins in Spanish-speaking countries. This group also comprises people who identify as Hispanic regardless of their racial background. Between 2022 and 2023, the Hispanic population accounted for nearly 72% of the overall growth of the United States population, driven primarily by Hispanic births and decrease in death rates, according to the Vintage 2023 Population Estimates from the U.S. Census Bureau [1]. Hispanics of any race grew to just over 65 million, an increase of 1.16 million (1.8%) from the prior year [1]. This growth significantly contributed to the nation's total population gain of 1.64 million in 2023 [1]. Physicians practicing in the United States can therefore expect to see an increasing number of Hispanic patients in the years to come and should become familiar with the potential challenges involved in diagnosing and treating this population. According to the National Center for Health Statistics, in 2022 about 14.5% of Hispanic adults experienced symptoms of anxiety in the past two weeks compared to 16.5% of non‑Hispanic White adults [2]. Efforts to provide effective mental health care can be complicated by social perceptions surrounding mental illness in many Hispanic communities, which can lead to underreporting of symptoms, delays in seeking care, and reliance on somatic language to express psychological distress. Recognizing and incorporating culturally specific idioms of distress may provide a less stigmatizing framework for patients to describe their symptoms. This approach can help clinicians engage Hispanic patients in discussions about mental health while reducing barriers created by negative perceptions of psychiatric labels.

Treating this population can present cultural challenges, especially when it comes to provision of mental health services. "El salto" is a culturally recognized physical sensation associated with anxiety and emotional distress in Spanish-speaking Caribbean communities. Although literature describing this term is limited, it is commonly used in these communities, primarily in middle-aged and older individuals. Described as a sudden, involuntary physical sensation felt in the upper abdomen or chest, “el salto” is a sensation often associated with anxiety, emotional distress, or spiritual imbalance that reflects a culturally embedded way of experiencing and expressing discomfort. Although not formally recognized in the Diagnostic and Statistical Manual of Mental Disorders, Fifth Edition (DSM-5), "el salto" can be thought to parallel established Hispanic idioms like* ataque de nervios, nervios,* and *susto, *which are linked to anxiety and mood disorders and characterized by somatic and affective manifestations following psychosocial stressors [3,4]. 

Understanding and recognizing "el salto" within its cultural context is crucial for developing effective, culturally informed treatment strategies that bridge traditional beliefs with psychiatric practices. This case of “el salto” exemplifies the complexities of diagnosing anxiety when symptoms differ from conventional presentations and include a cultural component, highlighting the need for cultural competency in clinical practice. Behavioral healthcare poses a distinct set of challenges, as it often requires navigating culturally specific idioms of distress, differing help-seeking behaviors, and stigma that are not as prevalent in other areas of medicine.

## Case presentation

An 87-year-old Spanish-speaking woman of Cuban origin with a recent diagnosis of Parkinson's disease (PD) presented to the clinic for the first time due to a sad mood. Since her diagnosis of PD three months prior to the visit, she reported feeling down most days. Her daughter encouraged her to visit the psychiatrist. During those three months, the patient also reported difficulties with waking up in the morning and no longer found enjoyment in activities she used to participate in, such as knitting and calling friends. She endorsed low energy and decreased motivation to complete her daily activities. She had been sleeping an average of nine hours a night interrupted by three to four awakenings, during which she would stay awake for some time worrying about her family and then fall back asleep within one to two hours. These sleeping habits began a few years prior to the visit but worsened within the three months prior to the visit. She denied any current or previous suicidal ideation or attempt or manic episodes. 

The patient also endorsed worsening anxiety in the afternoons, during which she described "un salto", or "a jump", in her abdomen. To explore further, we used the Cultural Formulation Interview (CFI) as a guideline in this interview, which provided a structured open-ended framework for exploring the patient’s cultural identity, values, and explanatory model for distress [5]. The CFI guides practitioners in understanding how cultural identity, values, explanatory models, and social context influence symptom expression, help-seeking behavior, and treatment preferences [5]. The CFI draws on Arthur Kleinman’s concept of the Explanatory Model of Distress (EMD), which emphasizes understanding how patients perceive the cause, meaning, and impact of their symptoms. This model seeks to elicit the patient’s own framework for illness, including cultural identity, explanatory beliefs, stressors and supports, and preferences for care, so that clinicians can align treatment with the individual’s cultural context [5]. In our assessment, we followed these principles to clarify how the patient described “el salto” as an expression of anxiety. When asked to explain the sensation, she described the feeling "como una revoltura en el estómago" which translates to "like a churning in the stomach". She reported it sometimes felt as if food didn't settle well in her stomach but only for brief periods, specifically during times of distress or worry. This was not a new symptom for her, however. The patient reported experiencing this sensation since her teenage years during times of nervousness. Born and raised in Cuba, she confirmed that most women from her country commonly experience this sensation. She recalled learning about “el salto” for the first time when she was young because both her mother and grandmother experienced it. However, this “salto” was not why she presented to the visit. She stated that this symptom has been a "normal" part of her life for many years, as it is for so many that share her culture, and does not necessarily require medical attention. The primary reason for this visit was the newer depressive symptoms that started after her PD diagnosis. She did not believe one needs to have an anxiety diagnosis to experience “el salto”, but agrees that most people in her culture who may have been diagnosed with a psychological condition probably have experienced it. For the past nine years she has had trouble sleeping and has worried about several aspects of life such as the success of her children, her own health, her husband’s health, her future, and catastrophic events of the world. She has found it difficult to control these worries, but alprazolam has helped her sleep slightly better during the night.

Past medical history

The patient has had no previous psychiatric diagnoses, psychiatric hospitalizations, or use of any psychotropic drugs except for alprazolam. Despite alprazolam use, she denied previous anxiety diagnosis. Her past medical history consists of hypertension, insomnia, and Parkinson's disease. Her medications at the time of the visit were amlodipine 2.5 mg once daily, losartan 50 mg once daily, carbidopa 10 mg-levodopa 100 mg three times daily, carbidopa 25 mg-levodopa 100 mg disintegrating tablets two tablets four times daily, and alprazolam 0.5 mg once nightly. The alprazolam was prescribed by her primary care physician eight years prior to her visit for insomnia. She has never had any surgeries and was hospitalized once at 82 with pneumonia. 

Psychosocial history

The patient was born in Cuba and lived there until her 30s. She married her husband in Cuba and later moved to Miami with two small children. The patient was a secretary at a legal office and retired at the age of 68 years. She has been living with her husband in a one-story house. Her two children are grown and healthy. They visit her often and have been supportive. They are financially stable and since her PD diagnosis, a Home Health Aid has been visiting her home for two hours daily to help her bathe. The patient and her husband have been attending church weekly, and she has volunteered at her church occasionally. She previously enjoyed knitting clothing and blankets for her grandchildren but had not done so for the three months prior to the visit. She has also stopped speaking on the phone to her friends from church. Her diet has consisted of Cuban foods such as steak, chicken, pork and rice and beans. She endorsed a slightly decreased appetite. She does not exercise but ambulates in her house with the help of a walker to complete household tasks. She uses a wheelchair for doctor's appointments and other outside events. She does not drive but her husband does. She denies alcohol use, recreational drug use, and has never smoked. The patient has not been sexually active for the past three years and attributes this to age. 

Lab history

The patient met the DSM-5 criteria for both generalized anxiety disorder (GAD) and major depressive disorder (MDD) [6]. Her comprehensive blood count, comprehensive metabolic panel, vitamin B12, and thyroid-stimulating hormone done three months prior to the visit with her neurologist were within range, ruling out some medical causes of depressive symptoms such as vitamin deficiency and thyroid pathology. To rule out other alternative medical explanations, gastrointestinal, cardiac, and neurologic causes were considered. Her symptoms did not follow any post-prandial pattern, had no associated nausea, vomiting, or changes in bowel habits, and lacked the persistent quality typical of gastrointestinal pathology. Cardiac causes such as arrhythmia or angina were unlikely given the absence of chest pain, palpitations, or exertional triggers. EKG conducted at her primary care physician’s office was unremarkable. Neurologic contributors such as PD-related autonomic dysfunction were also considered, but she reports feeling this “salto” since she was young. However, PD-related dysfunction could play a role in her depressive symptoms. 

Mental status examination

Upon mental status examination her appearance was well-groomed, clean, and she appeared of stated age. She was cooperative, calm, and maintained eye contact. Her speech was fluent and clear. No hallucinations or internal preoccupations noted. She was alert and oriented to situation, time, and place. Intelligence was average and insight was intact. Her mood was sad and affect was tearful. Judgment and thought process were intact, and her motor activity was limited due to age and PD. Her Patient Health Questionnaire‑9 (PHQ-9) score was 12, consistent with moderate depression and her General Anxiety Disorder‑7 (GAD-7) was 11, consistent with moderate anxiety [7,8]. 

Current history

The temporal association between her PD diagnosis and the exacerbation of depressive and anxious symptoms suggests that both the physiological changes of PD and the psychosocial stress of the diagnosis likely played a role in her mood disturbance. The emotional impact of receiving a life-altering diagnosis such as PD can be significant. On the other hand, depression and anxiety in PD can also be linked to the degeneration of dopaminergic, serotonergic, and noradrenergic systems. Studies have shown that serotonergic degeneration, particularly in the subgenual anterior cingulate cortex and orbitofrontal cortex, plays a significant role in the development of these symptoms [9,10]. Additionally, disruptions in cortico-limbic, striato-thalamic-prefrontal, and mediotemporal-limbic networks may contribute to mood disturbances [11]. Because of this, the patient’s anxiety and depression can be conceptualized as arising from an interplay between both the neurobiological impact of PD and the psychological response to chronic illness. Antidepressants have been found to be effective in PD patients with depression and anxiety, so this option was presented to the patient [12]. After discussing treatment options, the patient agreed to start escitalopram 5 mg once daily for anxious and depressive symptoms as well as bupropion extended release 150 mg once daily for the worsening depressive symptoms. Though it was recommended to gradually discontinue the alprazolam due to age-related adverse effects such as cognitive impairment and increased risk for falls, the patient declined to discontinue the medication at that time. She also declined the recommendation for psychotherapy but agreed to return for progress monitoring three weeks after the visit.

## Discussion

This case study analyzes a patient who describes a somatic symptom commonly known in the Hispanic Caribbean community as “el salto”. There appears to be a complex interplay between cultural and psychiatric themes in this case, and it is imperative to understand the significance of these within the patient’s unique environment. The principle of linguistic relativity, which is the idea that the language we speak shapes how we perceive and think about our experiences, can help explain why our patient has long understood her anxiety primarily as the somatic “jump” of “el salto” rather than as an emotional or psychiatric phenomenon. In many Hispanic communities, idioms such as *nervios, ataques de nervios, *and* susto *serve as culturally embedded frameworks for distress, privileging physical sensations over affective states and often steering patients toward medical rather than psychological care [3,4]. This could contribute to the underreporting of anxiety and depression in clinical settings, as patients may describe symptoms in terms of gastrointestinal distress, cardiovascular sensations, or other bodily complaints rather than psychological distress. In this case, the patient’s linguistic and cultural schema led her to underrecognize her symptoms as part of a treatable anxiety or depressive disorder, delaying psychiatric intervention until her explanatory model was explicitly explored. 

The importance of cultural competence is highlighted when discussing “el salto”, as it may lead to a more culturally sensitive way of diagnosing and describing psychiatric conditions such as GAD. According to the DSM-5, a diagnostic criterion for GAD includes physical symptoms causing distress in social, occupational, or other areas of function [8]. “El salto” should be recognized as a physical symptom closely associated with anxiety, although it is unclear if the patients who utilize this term are aware of what it may mean for their diagnosis. Helping patients identify the clinical significance of this symptom may enable a broader population of patients to receive the care they need. 

A key strength of this case report is its in‑depth, patient‑centered exploration of “el salto” using the Cultural Formulation Interview, which illuminates how a long‑standing, culturally grounded somatic idiom can shape a patient’s understanding of anxiety and guide care. For instance, when asked about possible causes (CFI Q4), she explained that the “salto” in her stomach emerged during periods of family-related worry, framing it as both a physical and emotional experience. Similarly, when asked whether any aspects of her background or identity affected her problem (CFI Q9), she noted that in her community, people often talk about "nervios" or a "salto" when describing the body’s reaction to stress, which validated her interpretation of the experience. These insights allowed the clinician to recognize “el salto” as a culturally rooted idiom rather than a nonspecific somatic complaint. Another strength was situating “el salto” alongside well‑described Hispanic syndromes such as *nervios *and *susto,* which provides a clear framework for clinicians to recognize and integrate this idiom into psychiatric assessment. However, because we reflect only one individual’s experience, our findings cannot be assumed to generalize across diverse Hispanic or Caribbean communities. The patient’s retrospective descriptions may also be influenced by recall bias and by her own explanatory model, limiting the ability to separate cultural narrative from underlying psychopathology. Finally, without standardized measures of “el salto” or comparison to a larger sample, its prevalence, severity thresholds, or response to intervention cannot yet be determined. Future studies should validate “el salto” in broader cohorts, quantify its relationship to anxiety disorders, and develop culturally tailored screening tools.

Practical considerations and clinical strategies for achieving a more culturally attuned approach to anxiety can be seen in Table 1. These are basic techniques inspired from sources such as the DSM-5 CFI that can be applied in a clinical setting and aid physicians in identifying symptoms of distress that may be psychiatric in nature. Moving forward, there is much to be done in the exploration of cultural syndromes such as “el salto”. Utilizing culturally informed questioning rather than relying on traditional, and often unfamiliar, terms may improve the identification of psychiatric criteria. Establishing more culturally relevant diagnostic screening tools may also aid in establishing diagnoses as they reframe somatic symptoms into a psychiatric context. Additionally, future research should examine how different linguistic contexts, including bilingual settings, influence symptom reporting and diagnosis, so that we can refine culturally responsive approaches to anxiety across diverse populations. As the field of psychiatry evolves and our multicultural population grows, a nuanced understanding of culture-bound expressions of distress will be essential for ensuring that all patients, regardless of background, receive compassionate and competent care.

**Table 1:** Suggested Considerations for Clinicians: A Culturally Attuned Approach to Anxiety Table is created by the authors using information from Diagnostic and Statistical Manual of Mental Disorders, Fifth Edition (DSM-5) Cultural Formulation Interview [4].

Domain	Clinical Strategy	Rationale
Elicit Culturally Specific Symptoms	Use open-ended, culturally sensitive questions: - “Some people experience anxiety or distress in physical ways. Have you noticed any bodily sensations where you feel nervous or worried?” - “How would you describe what you're feeling to a close friend or family member?” - “Have you heard of others in your community experiencing something similar?”	Encourages patients to describe symptoms in their own terms, reducing reliance on rigid diagnostic criteria and surfacing idioms of distress like “el salto”.
Utilize DSM-5 Cultural Formulation Interview (CFI)	Employ the CFI to explore cultural context, explanatory models, and help-seeking behavior.	Clarifies whether symptoms are culture-bound or more universal; aligns diagnostic formulation and treatment planning with patients’ cultural values and beliefs.
Reframe Symptoms Without Dismissing Cultural Meaning	Validate the symptom while linking it to a psychiatric framework: - “It makes sense that you experience el salto during stress, we often see anxiety manifest physically.”	Respects cultural meaning while expanding clinical understanding, helping foster openness to psychiatric care and reducing potential resistance to diagnosis or treatment.
Bridge Cultural and Psychiatric Models	Integrate traditional and biomedical approaches: - Ask about traditional coping strategies. - Introduce psychoeducation gradually. - Tailor interventions to include cultural elements.	Enhances treatment adherence and engagement by positioning psychiatric care as complementary and not contradictory to patients' cultural worldviews and lived experiences.

Brief literature review: cultural idioms

*Ataque de nervios*, translating literally to "attack of the nerves" in English, involves intense emotional outbursts (crying, shouting, trembling) and dissociative features in response to stressors; it is strongly associated with anxiety, depressive, and dissociative disorders, affecting up to 15% of Puerto Rican adults and conferring four times greater odds of psychiatric morbidity compared to those who did not experience *ataque de nervios* [3,13]. *Nervios*, which literally translates to "nerves" in English, describes chronic dysphoric states with somatic complaints that are common among socially vulnerable groups, and correlate with depressive disorders and sleep disturbances [4,14]. *Susto *(fright) entails somatic and affective symptoms attributed to soul loss after a frightening event and is linked to depression and functional impairment, particularly in lower socioeconomic contexts [4,15]. Recognizing these idioms improves diagnostic accuracy by distinguishing culturally normative expressions of distress from pathologic presentations. Studies demonstrate that idioms like* ataque de nervios *and *susto *share etiological stressors and overlap with anxiety disorders, yet they are embedded in cultural meaning systems that influence symptom interpretation, help-seeking, and coping strategies [3,4,13]. Failure to recognize these idioms risks mislabeling culturally normative experiences as psychiatric conditions such as psychosis or conversion disorder.

Sheikh and Furnham (2012) conducted a cross-sectional investigation into how culture and demographic variables influence the somatic expression of psychological distress and general practitioner (GP) consultations among British Asians and native White Britons. Using the Bradford Somatic Inventory (BSI) and the General Health Questionnaire (GHQ-28), the study found that British Asians reported significantly higher levels of somatic symptoms, particularly among those with low English proficiency [16]. Somatic expression was closely linked to psychological distress and more frequent GP visits in the British Asian group, while this pattern was not observed in the White British group. Income, age, and language proficiency were also significant predictors of somatic symptom reporting [16]. These findings highlight the role of culture as a predictor of distress as a somatic expression. Demographics and socioeconomic status were also shown to be significant variables [16]. 

Noguera et al. (2009) conducted a prospective study to assess the effectiveness of different Spanish terms for screening for depression and anxiety in palliative care patients. They found that the term *desanimado* ("discouraged") had a stronger correlation with the Hospital Anxiety and Depression Scale (HADS) depression subscale and was preferred by patients over *deprimido* ("depressed") or* triste* ("sad") [17]. For anxiety, no single term - *ansioso *("anxious"), *nervioso* ("nervous"), or* intranquilo* ("uneasy") - was clearly favored, though all showed moderate correlation [17]. The study highlights that subtle linguistic variations in symptom descriptors can significantly impact patient responses and screening accuracy. These findings underscore the need for culturally and linguistically adapted tools in clinical assessments. This is particularly important in the context of Hispanic cultures, which have traditionally been hesitant towards psychiatric care and mental health. Within these cultural frameworks, individuals may be reluctant to endorse more severe or pathologizing terms such as *deprimido* [17]. In contrast, *desanimado* is a less intense, less stigmatized expression, which may make it more acceptable and approachable for patients [17]. As such, offering "less severe" or culturally softer language could serve as a valuable conduit into discussing psychiatric symptoms with hesitant Hispanic patients. The above findings emphasize the need for culturally and linguistically adapted tools in clinical assessments.

In their study, Dunlop et al. (2020) examined the prevalence of somatic symptoms among treatment-naïve patients with MDD, comparing Hispanic, non-Hispanic Black, and non-Hispanic White individuals. The researchers found that Hispanic patients, particularly those who spoke only Spanish, reported significantly higher levels of somatic symptoms than their non-Hispanic counterparts [18]. Interestingly, psychological anxiety symptoms did not vary significantly across ethnic groups, suggesting a specific cultural pattern in symptom expression. Language use, serving as a metric for acculturation, emerged as a key factor, with Spanish-speaking Hispanics exhibiting the highest somatic symptom scores [18]. These findings highlight that Hispanic patients, specifically those who speak only Spanish, are at higher likelihood of expressing somatic symptoms when compared to non-Hispanic counterparts.

Taken together, these findings emphasize the broader significance of cultural idioms of distress, and provide a foundation for integrating them into clinical assessment and care. Terms such as *ataque de nervios, nervios, susto, desanimado, *and *intranquilo* represent culturally resonant expressions of anxiety, depression, and somatic suffering that overlap with DSM-defined disorders, yet remain grounded in distinct sociocultural contexts. As summarized in Table 2, each idiom encompasses a unique constellation of symptoms and connotations that shape patient-clinician communication, diagnostic interpretation, and help-seeking behavior. Collectively, the reviewed studies emphasize the critical role of culturally and linguistically attuned assessments in improving diagnostic accuracy, reducing stigma, and fostering patient engagement in mental health care.

**Table 2:** Common Hispanic Idioms of Distress Table is created by the authors using information from Guarnaccia et al. [12], Durà-Vilà and Hodes [13], and Noguera et al. [16].

Idiom	Literal Translation	Core Features	Cultural Context	Associated Psychiatric Disorders
El Salto	The jump	Sudden abdominal churning during distress	Spanish-speaking Caribbean; seen as normative	Generalized Anxiety Disorder
Ataque de Nervios	Attack of the nerves	Emotional outbursts, dissociation in response to distress	Puerto Rican; 15% prevalence; high psychiatric comorbidity	Anxiety, Depression, Dissociative Disorders
Nervios	Nerves	Chronic dysphoria with somatic complaints	Common in vulnerable Hispanic populations	Depression, Sleep Disturbance
Susto	Fright	Somatic symptoms attributed to soul loss after fright	Widespread in Latin America; associated with traumatic stress	Depression, Functional Impairment
Desanimado	Discouraged	Low energy, sadness; preferred term over ‘depressed’	Used in Spanish-speaking palliative care to soften stigma	Major Depressive Disorder
Intranquilo	Uneasy	Mild unease or agitation; associated with anxiety	Alternative to ‘anxious’; moderate symptom language	Anxiety Disorders

## Conclusions

This case highlights the critical role of cultural competence in psychiatric evaluation and treatment. The presentation of "el salto" as a somatic expression of anxiety highlights the complexity of diagnosing mental health conditions in culturally diverse populations. By recognizing "el salto" not as a distinct medical condition but as a culturally embedded idiom of distress, clinicians can avoid misdiagnosis and ensure that patients receive care that is both effective and culturally attuned. 

More broadly, this case illustrates the importance of integrating cultural formulations into psychiatric practice. A deeper understanding of culture-bound syndromes like "el salto" can enhance diagnostic accuracy, improve patient-clinician communication, and foster trust in mental health care systems. As the Hispanic population in the United States continues to grow, so too does the need for culturally responsive approaches that acknowledge the intersection of traditional beliefs and modern psychiatry. 

Future research should explore the prevalence and clinical implications of "el salto" within Hispanic communities, particularly its potential role in the assessment of anxiety disorders. Furthermore, efforts to incorporate cultural idioms of distress into diagnostic frameworks could refine psychiatric evaluations and inform targeted interventions. By embracing a culturally informed perspective, mental health professionals can bridge the gap between biomedical psychiatry and the lived experiences of diverse patient populations, ultimately advancing more inclusive and effective mental health care.

## References

[1] (2023). QuickFacts United States. https://www.census.gov/quickfacts/fact/table/US/RHI725221.

[2] Terlizzi Terlizzi, E. P. (2020 (2020). Symptoms of generalized anxiety disorder among adults: United States, 2019. https://www.cdc.gov/nchs/products/databriefs/db378.htm.

[3] Moitra E, Duarte-Velez Y, Lewis-Fernández R, Weisberg RB, Keller MB (2018). Examination of ataque de nervios and ataque de nervios like events in a diverse sample of adults with anxiety disorders. Depress Anxiety.

[4] Guarnaccia PJ, Rivera Marano M, Martinez Pincay I, Lewis-Fernandez R (2002). The prevalence of ataque de nervios and their association with psychiatric diagnosis. Cult Med Psychiatry.

[5] (2013). Cultural Formulation Interview. Diagnostic and Statistical Manual of Mental Disorders (5th Edition).

[6] (2022). Diagnostic and statistical manual of mental disorders (5th. Diagnostic and Statistical Manual of Mental Disorders (5th Edition).

[7] Kroenke K, Spitzer RL, Williams JB (2001). The PHQ-9: validity of a brief depression severity measure. J Gen Intern Med.

[8] Spitzer RL, Kroenke K, Williams JB, Löwe B (2006). A brief measure for assessing generalized anxiety disorder: the GAD-7. Arch Intern Med.

[9] Maillet A, Krack P, Lhommée E (2016). The prominent role of serotonergic degeneration in apathy, anxiety and depression in de novo Parkinson's disease. Brain.

[10] Maillet A, Météreau E, Tremblay L (2021). Serotonergic and dopaminergic lesions underlying Parkinsonian neuropsychiatric signs. Mov Disord.

[11] Jellinger KA (2022). The pathobiological basis of depression in Parkinson disease: challenges and outlooks. J Neural Transm (Vienna).

[12] Wang XL, Feng ST, Wang YT, Chen B, Wang ZZ, Chen NH, Zhang Y (2022). Comparative efficacy and acceptability of drug treatments for Parkinson's disease with depression: a systematic review with network meta-analysis. Eur J Pharmacol.

[13] Guarnaccia PJ, Lewis-Fernandez R, Martinez Pincay I (2010). Ataque de nervios as a marker of social and psychiatric vulnerability: results from the NLAAS. Int J Soc Psychiatry.

[14] Durà-Vilà G, Hodes M (2012). Cross-cultural study of idioms of distress among Spanish nationals and Hispanic American migrants: susto, nervios and ataque de nervios. Soc Psychiatry Psychiatr Epidemiol.

[15] Weller SC, Baer RD, Garcia CA, Glazer M, Trotter RT, Klein RE (2002). Regional variation in Latino descriptions of susto. Cult Med Psychiatry.

[16] Sheikh S, Furnham A (2012). The relationship between somatic expression, psychological distress and GP consultation in two cultural groups. Coun Psychol Q.

[17] Noguera A, Centeno C, Carvajal A, Tejedor MA, Urdiroz J, Martínez M (2009). Spanish "fine tuning" of language to describe depression and anxiety. J Palliat Med.

[18] Dunlop BW, Still S, LoParo D (2020). Somatic symptoms in treatment-naïve Hispanic and non-Hispanic patients with major depression. Depress Anxiety.

